# *Candida* concentrations determined following concentrated oral rinse culture reflect clinical oral signs

**DOI:** 10.1186/s12903-015-0138-z

**Published:** 2015-11-24

**Authors:** Hiroaki Tooyama, Takehisa Matsumoto, Kiyonori Hayashi, Kenji Kurashina, Hiroshi Kurita, Mitsuo Uchida, Eriko Kasuga, Takayuki Honda

**Affiliations:** Department of Dentistry and Oral Surgery, Shinshu University School of Medicine, Shinshu University Hospital, 3-1-1 Asahi, Matsumoto, 390-8621 Japan; Department of Laboratory Medicine, Shinshu University School of Medicine, Shinshu University Hospital, 3-1-1 Asahi, Matsumoto, 390-8621 Japan; Department of Dentistry and Oral Surgery, Aizawa Hospital, 2-5-1 Honjo, Matsumoto, 390-8510 Japan; Center for Health, Safety and Environmental Management, Shinshu University, 3-1-1 Asahi, Matsumoto, 390-8621 Japan

**Keywords:** Concentrated oral rinse method, Swab method, Oral candidiasis, *Candida*

## Abstract

**Background:**

Oral candidiasis is an infection caused by a yeast-like fungus called *Candida*. Various methods can be used to isolate *Candida* from the oral cavity. However, it is difficult to correctly and satisfactorily diagnose oral candidiasis because currently no microbiological or laboratory standards based on samples from the oral cavity are available. The aim of this study is to establish a reliable laboratory test for diagnosing oral candidiasis.

**Methods:**

Oral swab, rinse and concentrated rinse samples were obtained from 200 consecutive outpatients (103 male patients and 97 female patients; mean age, 47.2 years; age range, 9–89 years). *Candida* colonies from cultured samples were enumerated to compare the sensitivities and specificities of the above sampling methods, and the associations between *Candida* detection or concentration and the clinical oral signs were examined.

**Results:**

The mean colony numbers were 263 ± 590 CFU/swab for the swab method, 2894 ± 6705 CFU/100 μL for the rinse method, and 9245 ± 19,030 CFU/100 μL for the concentrated rinse method. The median numbers were 23 CFU/swab for the swab method, 56 CFU/100 μL for the rinse method, and 485 CFU/100 μL for the concentrated rinse method. *Candida* was detected in the oral cavity of 33.5 % and 52.0 % of the outpatients by the swab method and concentrated rinse, respectively. *Candida* concentrations determined by the concentrated rinse were closely related to the severity of the clinical oral signs. The positive predictive values of residual root, redness of the oral mucosa, denture, glossalgia, dry mouth, and taste disorder were useful predictors of oral candidiasis.

**Conclusions:**

Concentrated rinse sampling is suitable for evaluating oral candidiasis, and *Candida* concentrations examined using this method strongly associated with the oral signs associated with *Candida* infection.

## Background

Oral candidiasis is a common opportunistic infection of the oral cavity and is caused by yeast of the *Candida* genus, primarily *Candida albicans*. It presents clinically in many forms, including pseudomembranous (acute/chronic), erythematous (acute/chronic), plaque-like (chronic), and nodular (chronic) forms [1]. However, *Candida* species are frequently isolated from the oral cavity in healthy individuals of all ages, with a reported prevalence of 15–75 % [2–4], and it is therefore difficult to differentiate oral candidiasis from the commensal state by microbiological detection of the *Candida* species in the oral cavity. Furthermore, oral candidiasis has often been diagnosed on the basis of clinical findings, regardless of whether a *Candida* species was detected. Therefore, additional microbiological criteria are required to diagnose oral *Candida* infection correctly.

Various methods can be used to isolate *Candida* from the oral cavity, including smears, plain swabs, imprint cultures, whole saliva collection, concentrated oral rinses, and mucosal biopsies [5, 6]. Of these, the concentrated oral rinse method is one of the most suitable techniques for determining *Candida* concentrations in the oral cavity [7]; however, this method is inadequate for detecting the *Candida* infection site. *Candida* concentrations under 600 CFU/mL in concentrated rinse samples have been reported for healthy commensal carriage [8], whereas individuals with *Candida* concentrations above 2–3 × 10^3^ CFU/mL are predisposed to oral *Candida* infection [7]. However, White et al. reported that *Candida* levels up to 9 × 10^3^ CFU/mL were observed in healthy controls and that these levels were occasionally higher than those in patients with oral candidiasis [9].

Oral candidiasis frequently occurs in immunocompromised individuals, including HIV-positive and AIDS patients, organ transplant recipients, and chemotherapy patients [10]. In fact, the disease is often the initial sign of several immunodeficiency diseases, and its clinical significance as a biomarker has been recognized in recent years [11]. However, it is difficult to correctly and satisfactorily diagnose oral candidiasis because currently no microbiological or laboratory standards based on samples from the oral cavity are available. In this study, we examined associations between clinical oral findings and difference methods for obtaining samples from the oral cavity to determine which criteria could help differentiate oral candidiasis from the presence of *Candida* in the commensal state.

## Methods

Samples obtained from 200 consecutive outpatients (103 male patients and 97 female patients; mean age, 47.2 years; age range, 9–89 years) who consulted a dentist at Aizawa Hospital from March 2011 to June 2011 were participated in this study. Samples from 30 volunteers (17 men and 13 women; mean age, 30.1 years; age range, 23–43 years) without clinical oral symptoms and signs of candidiasis were also used. In all of them, one tooth was not broken and the decayed teeth were completely treated. Informed consent was obtained from all patients, the parents of minors, and volunteers. The Committee for Ethics at Aizawa Hospital approved this study protocol with approval number H22-14.

### Sample preparation and determination of CFU

The 3 sample methods used in the study were as follows.

*1. Swab method*: The materials were obtained by swabbing the dorsal surface of the tongue with 5 strokes (about 2 cm in length) of a cotton swab (Hakujuji Co Ltd. Tokyo, Japan), and then the swab was directly inoculated onto CHROMagar Candida medium (Kanto Chemical Co. Ltd., Tokyo, Japan).

*2. Rinse method*: After a sample had been obtained using the swab method, a sample of oral rinse solution was collected by rinsing the mouth with 10 mL sterile saline, which was held in the mouth for 5 s before being collected in a sterile container. One hundred microliters of the rinse solution was inoculated onto the CHROMagar Candida medium.

*3. Concentrated rinse method*: The oral cavity is rinsed with 10 mL of sterile saline, and 7 to 10 mL was collected as the rinse solution. The concentrated rinse solution was prepared by centrifuging it at 2300 × *g* for 20 min. After the supernatant was removed, the cell pellet was resuspended in 500 μL, which was inoculated onto CHROMagar Candida medium in 100 μL aliquots. *Candida* colonies were counted after incubation at 37 °C for 48 h. If there were too many *Candida* colonies to be counted, the *Candida* solutions were diluted tenfold.

### Associations between the presence of *Candida* species and clinical oral signs

We then examined associations between the presence of *Candida* species and clinical oral signs using samples obtained via the swab method and the concentrated rinse method. Associations between *Candida* colony counts (*Candida* concentrations) and clinical oral signs were then determined using samples obtained via the concentrated rinse method. Table 1 shows the clinical oral signs used in this study and their grading.

**Table 1:** Clinical oral signs and their grading

	Grade			
Signs	0	1	2	3
Glossalgia	Negative	Slight	Moderate	Severe
Taste disorder	Negative	Slight	Moderate	Severe
Dry mouth	Negative	Slight	Moderate	Severe
Redness of oral mucosa	Negative	Slight	Moderate	Severe
Redness of the tongue	Negative	Slight	Moderate	Severe
Coated tongue	Negative	Slight	Moderate	Severe
Angular cheilitis	Negative	Unilateral	Bilateral	
Ulceration	Negative	Single	Multiple	
Residual root	Negative	Single	Multiple	
Denture	Negative	Unilateral	Bilateral	

### Oral assessments

Clinical oral signs were graded as follows. Glossalgia was graded using the Visual Analog Scale (negative: 0 mm; slight: 1 mm; moderate: 30 mm; severe: over 54 mm) [12, 13]. Taste disorder was graded using the Common Terminology Criteria for Adverse Events v3.0 published by the National Cancer Institute (negative: no change in taste; slight: altered taste but no change in diet; moderate: altered taste with change in diet or noxious or unpleasant taste; severe: loss of taste) [14]. Dry mouth was graded using the classification provided by Kakinoki et al. (negative: non-dry; slight: saliva shows viscosity; moderate: saliva showing tiny bubbles on tongue; severe: dry tongue without viscosity, little or no saliva) [15]. Redness of oral mucosa was graded using the Eilers Oral Assessment Guide (negative: no redness on the oral mucosa; slight: localized redness areas without ulcerations; moderate: redness on the whole oral mucosa without ulcerations; severe: ulcerations with or without bleeding) [16].

Tongue coating was graded using the visual scores developed by Kojima et al. (negative: less than 1/3 of the tongue slightly coated; slight: about 2/3 of the tongue slightly coated or about 1/3 of the tongue thickly coated; moderate: about 2/3 of the tongue thickly coated; severe: more than 2/3 of the tongue thickly coated [17]. Redness of the tongue was graded similarly (negative: less than 1/3 of the tongue showing slight redness; slight: about 2/3 of the tongue showing slight redness or about 1/3 of the tongue showing strong redness; moderate: about 2/3 of the tongue showing strong redness; severe: more than 2/3 of the tongue showing strong redness).

### Determining the normal range of healthy commensal carriage

We examined 30 volunteers without clinical oral signs of candidiasis for the presence of *Candida* species. We used the highest colony count obtained from their swab and concentrated rinse samples as the threshold for distinguishing oral candidiasis from the oral commensal state of *Candida* species. The *Candida* detection ratio, the associations between clinical oral signs and *Candida* detection, and the associations between clinical signs and the number of *Candida* colonies obtained using the swab method and the concentrated rinse method were then determined. The sensitivity and specificity of each clinical sign were examined when *Candida* species were detected.

### Statistical analysis

The *χ*2 test was used to determine the significance of the difference between the rates of positive *Candida* detection using the oral swab method and the concentrated oral rinse method. The median values of the number of detected *Candida* which were obtained from identical individuals were compared using the non-parametric Wilcoxon signed rank test. The significance of the relationships between the median *Candida* concentrations and the grades of each clinical oral sign was analyzed using the nonparametric Kruskal-Wallis test.

Statistical significance was set at *p* < 0.05 for all the analysis methods. In addition, Bonferroni test was used to adopt multiple comparison. All statistical analyses were performed using the SPSS software version 22 (SPSS, Chicago, IL, USA).

## Results

**Fig. 1:**
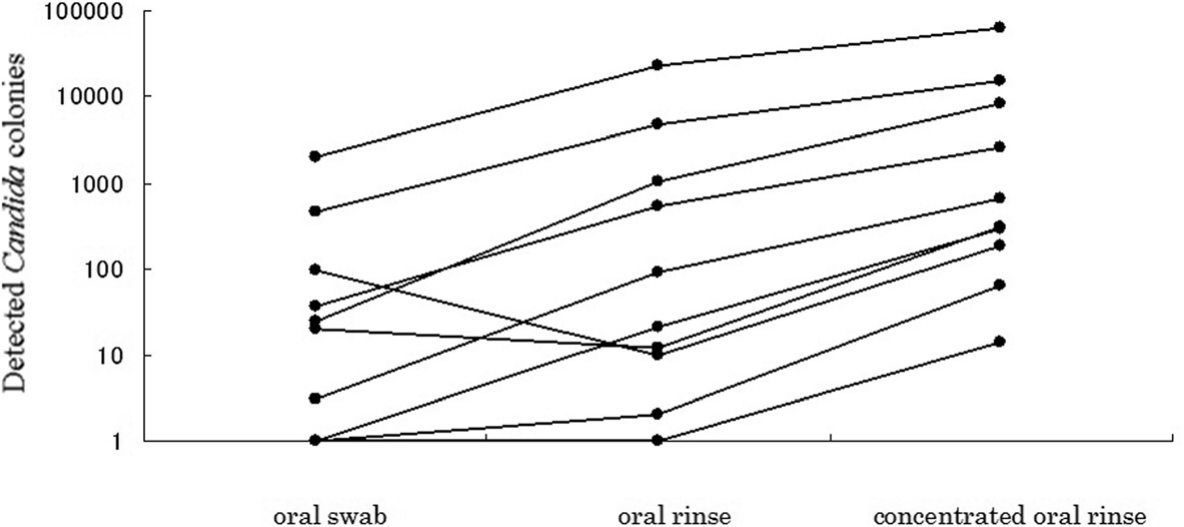
Comparison of the sensitivities of the swab method, the rinse method, and the concentrated rinse method (*n* = 10). Dots representing data from the same patient are connected by lines

In order to establish the required methods before the whole analysis, a pilot test was conducted on the first 10 samples. The colony counts obtained from the first 10 outpatients using the swab, rinse, and concentrated rinse methods are shown in Fig. 1. The median and interquartile range were 23 CFU (interquartile range, 3 to 96 CFU)/swab for the swab method, 56 CFU (interquartile range, 11 to 900 CFU)/100 μL for the rinse method, and 485 CFU (interquartile range, 210 to 6981 CFU)/100 μL for the concentrated rinse method in the first 10 outpatients. The first 10 outpatients were tested using all three methods; however, we used the concentrated rinse method for subsequent examinations because it yielded more *Candida* colonies. The median counts of the *Candida* colonies obtained using the concentrated rinse method were significantly higher than those obtained using the other two methods, respectively (*p* < 0.01, Wilcoxon signed-rank test with Bonferroni test). The concentrated rinse method was the most sensitive, because it could detect *Candida* species when the swab method or the rinse method did not. Thus, we understood that the concentrated rinse method was appropriate for subsequent examinations.

We presumptively identified *Candida* species from the color of colonies grown on CHROMagar Candida. Using this method, the following *Candida* profiles were observed in 68 patients, 12 patients, one patient, nine patients, seven patients, five patients, one patient, and one patient, respectively: *C. albicans*, *C. tropicalis*, *C. glabrata*, *C. albicans* + *C. glabrata*, *C. albicans* + *C. tropicalis*, *C. albicans* + *C. glabrata* + *C. tropicalis*, *C. albicans* + *C. krusei* + unidentified *Candida* species, and *C. glabrata* + *C. tropicalis* + unidentified *Candida* species. There were no significant differences in clinical oral signs between the 68 patients with *C. albicans* and the 12 with *C. tropicalis*.

Detection rates and colony counts obtained using the swab method and the concentrated oral rinse method are shown in Table 2. *Candida* species were detected in the oral cavity in 67 of 200 patients (33.5 %) by the swab method and in 104 of 200 (52 %) by the concentrated rinse method. The median colony count was 7 CFU (interquartile range, 2 to 37 CFU)/swab for the swab method and 141 CFU (interquartile range, 14 to 1001 CFU)/100 μL for the concentrated rinse method. The detection ratios (*p* < 0.01, *χ*^2^ test) and colony counts (*p* < 0.01, Wilcoxon signed-rank test) obtained using the concentrated rinse method were significantly higher than those obtained using the swab method.

**Table 2:**
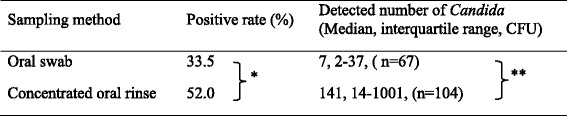
Detection rates and colony counts for the swab and concentrated rinse samples from patients (*n* = 200)

*χ ^2^ test (*p* < 0.01); **Wilcoxon signed-rank test (*p* < 0.01)

Associations between clinical oral signs and *Candida* detection using the swab method are summarized in Tables 3 and 4. When *Candida* was detected using the swab method, the sensitivities of coated tongue, dry mouth, denture, redness of the tongue, and residual root were 41.8, 46.3, 40.3, 34.3, and 29.9 %, respectively, and the specificities of redness of the oral mucosa, angular cheilitis, residual root, glossalgia, taste disorder, denture, and ulceration were 97.7, 99.2, 92.5, 94.0, 93.2, 88.0, and 92.5 %, respectively. The positive predictive values of residual root, redness of the oral mucosa, denture, glossalgia, dry mouth, and taste disorder were 66.7, 66.7, 62.8, 52.9, 54.4, and 35.7 %, respectively.

**Table 3:** Indices of clinical oral signs and detection of *Candida* by the swab method

Association between clinical oral signs and detection of *Candida* by the swab method				
		*Candida*		*P*-value
Clinical oral signs		(+)	(−)	(*χ* ^2^ test)
Glossalgia	(+)	9	8	0.08
	(−)	58	125	
Taste disorder	(+)	5	9	0.85
	(−)	62	124	
Dry mouth	(+)	31	26	<0.01
	(−)	36	107	
Redness of oral mucosa	(+)	6	3	<0.05
	(−)	61	130	
Redness of the tongue	(+)	23	25	<0.05
	(−)	44	108	
Coated tongue	(+)	28	42	0.15
	(−)	39	91	
Angular cheilitis	(+)	2	1	0.22
	(−)	65	132	
Ulceration	(+)	5	10	0.98
	(−)	62	123	
Residual root	(+)	20	10	<0.01
	(−)	47	123	
Denture	(+)	27	16	<0.01
	(−)	40	117

**Table 4:** Indices of clinical oral signs and detection of *Candida* by the swab method

Sensitivity, specificity, and positive predictive value between clinical oral signs and detection of *Candida* by the swab method			
Clinical oral signs	Sensitivity	Specificity	Positive predictive value
Glossalgia	13.4 %	94.0 %	52.9 %
Taste disorder	7.5 %	93.2 %	35.7 %
Dry mouth	46.3 %	80.5 %	54.4 %
Redness of oral mucosa	9.0 %	97.7 %	66.7 %
Redness of the tongue	34.3 %	81.2 %	47.9 %
Coated tongue	41.8 %	68.4 %	40.0 %
Angular cheilitis	3.0 %	99.2 %	66.7 %
Ulceration	7.5 %	92.5 %	33.3 %
Residual root	29.9 %	92.5 %	66.7 %
Denture	40.3	88.0 %	62.8 %

Associations between clinical oral signs and *Candida* detection using the concentrated rinse method are summarized in Tables 5 and 6. When *Candida* was detected using the concentrated rinse method, the sensitivities of coated tongue, dry mouth, denture, redness of the tongue, and residual root were 45.2, 42.3, 34.6, 29.8, and 26.0 %, respectively, and the specificities of redness of the oral mucosa, angular cheilitis, residual root, glossalgia, taste disorder, denture, and ulceration were 99.0, 99.0, 96.9, 96.9, 95.8, 92.7, and 91.7 %, respectively. The positive predictive values of residual root, redness of the oral mucosa, denture, glossalgia, dry mouth, and taste disorder were 90.0, 88.9, 83.7, 82.4, 77.2, and 71.4 %, respectively.

**Table 5:** Indices of clinical oral signs and detection of *Candida* by the concentrated rinse method

Association between clinical oral signs and detection of *Candida* by the concentrated rinse method				
		*Candida*		*P*-value
Clinical oral signs		(+)	(−)	(*χ* ^2^ test)
Glossalgia	(+)	14	3	<0.01
	(−)	90	93	
Taste disorder	(+)	10	4	0.13
	(−)	94	92	
Dry mouth	(+)	44	13	<0.01
	(−)	60	83	
Redness of oral mucosa	(+)	8	1	<0.05
	(−)	96	95	
Redness of the tongue	(+)	31	17	<0.05
	(−)	73	79	
Coated tongue	(+)	47	23	<0.01
	(−)	57	73	
Angular cheilitis	(+)	2	1	0.60
	(−)	102	95	
Ulceration	(+)	7	8	0.66
	(−)	97	88	
Residual root	(+)	27	3	<0.01
	(−)	77	93	
Denture	(+)	36	7	<0.01
	(−)	68	89

**Table 6:** Indices of clinical oral signs and detection of *Candida* by the concentrated rinse method

Sensitivity, specificity, and positive predictive value between clinical oral signs and detection of *Candida* by the concentrated rinse method			
Clinical oral signs	Sensitivity	Specificity	Positive predictive value
Glossalgia	13.5 %	96.9 %	82.4 %
Taste disorder	9.6 %	95.8 %	71.4 %
Dry mouth	42.3 %	86.5 %	77.2 %
Redness of oral mucosa	7.7 %	99.0 %	88.9 %
Redness of the tongue	29.8 %	82.3 %	64.6 %
Coated tongue	45.2 %	76.0 %	67.1 %
Angular cheilitis	1.9 %	99.0 %	66.7 %
Ulceration	6.7 %	91.7 %	46.7 %
Residual root	26.0 %	96.9 %	90.0 %
Denture	34.6 %	92.7 %	83.7 %

Differences between the grades of each clinical oral sign and colony numbers obtained using the swab method are shown in Table 7. High *Candida* counts were significantly associated with dry mouth. Differences between the grades of each clinical oral sign and colony concentrations obtained using the concentrated rinse method are shown in Table 8. High *Candida* counts were significantly associated with dry mouth, redness of the tongue, coated tongue, and denture.

**Table 7:** Differences between the grades of each clinical oral sign and *Candida* numbers by the swab method

Clinical oral signs		Grade				
		0	1	2	3	P*
Glossalgia	Median^a^	0	1	0	6.5	0.068
	(*n*)	183	9	6	2	
Taste disorder	Median	0	0	0	116	0.282
	(*n*)	186	9	4	1	
Dry mouth	Median	0	0.5	6	98	<0.001
	(*n*)	143	36	20	1	
Redness of oral mucosa	Median	0	24	0	0	0.015
	(*n*)	191	8	1	0	
Redness of the tongue	Median	0	0	36.5	0	0.002
	(*n*)	152	40	8	0	
Coated tongue	Median	0	0	0	7	0.138
	(*n*)	130	57	12	1	
Angular cheilitis	Median	0	58	3	NA^b^	0.402
	(*n*)	197	2	1	NA	
Ulceration	Median	0	0	0	NA	0.995
	(*n*)	185	11	4	NA	
Residual root	Median	0	6	5	NA	<0.001
	(*n*)	170	15	15	NA	
Denture	Median	0	0	5	NA	<0.001
	(*n*)	157	14	29	NA	

*Kruskal-Wallis test

^a^Median of *Candida* numbers for every grade in a clinical oral sign

^b^ *NA* not applicable

**Table 8:** Differences between the grades of each clinical oral sign and *Candida* concentrations by the concentrated rinse method

Clinical oral signs		Grade				
		0	1	2	3	P*
Glossalgia	Median^a^	0	137	1.5	1500.5	0.004
	(*n*)	183	9	6	2	
Taste disorder	Median	1	4	142.5	10000	0.163
	(*n*)	186	9	4	1	
Dry mouth	Median	0	127	269.5	1592	<0.001
	(*n*)	143	36	20	1	
Redness of oral mucosa	Median	1	1186	121	0	0.008
	(*n*)	191	8	1	0	
Redness of the tongue	Median	0	3	791	0	0.006
	(*n*)	152	40	8	0	
Coated tongue	Median	0	7	22	1520	0.037
	(*n*)	130	57	12	1	
Angular cheilitis	Median	1	5000	578	NA^b^	0.417
	(*n*)	197	2	1	NA	
Ulceration	Median	1	1	0	NA	0.710
	(*n*)	185	11	4	NA	
Residual root	Median	0	275	254	NA	<0.001
	(*n*)	170	15	15	NA	
Denture	Median	0	3	578	NA	<0.001
	(*n*)	157	14	29	NA	

*Kruskal-Wallis test

^a^Median of *Candida* concentrations for every grade in a clinical oral sign

^b^ *NA* not applicable

When *Candida* counts were determined in healthy volunteers, the swab method yielded colonies for 3/30 of the volunteers (1, 4, and 5 colonies, respectively), whereas the concentrated rinse method yielded colonies for 8/30 volunteers (1, 1, 2, 22, 25, 36, 38, and 67 colonies, respectively). Based on these results, we defined 0–5 CFU/swab and 0–67 CFU/100 μL as the reference ranges for healthy commensal carriages detected by the swab method and the concentrated rinse method, respectively. In contrast, among outpatients with no clinical oral signs, the highest counts obtained using the swab method and the concentrated rinse method were 23 CFU/swab and 90 CFU/100 μL, respectively.

## Discussion

In this study, *Candida* species were detected in the oral cavity in dental clinic outpatients with a frequency of 52.0 and 33.5 % using the concentrated rinse method and the swab method, respectively. Therefore, the concentrated rinse method was more sensitive than the swab method for detecting *Candida* species in the oral cavity. Some of the oral clinical signs (e.g., coated tongue, dry mouth, denture, redness of the tongue, and residual root) were relatively robust predictors for oral candidiasis. However, the positive predictive values of residual root, redness of the oral mucosa, denture, glossalgia, dry mouth, and taste disorder were high, and only these clinical oral signs were frequently associated with the presence of *Candida* species.

The concentrated rinse method is more suitable for the detection of *Candida* species in the oral cavity than the swab method. However, the number of colonies in the concentrated rinse samples was smaller than the theoretically predicted value of a 20-fold increase in the rinse samples. This might be related to the low centrifugal force of 2300 × *g*. In addition, the concentrated rinse method showed the same sensitivity as the rinse method when high numbers of colonies were present; however, the concentrated rinse method was more sensitive when only a few colonies could be obtained from the sample. For the first 10 outpatients examined in this study, the concentrated rinse method yielded more *Candida* colonies than the standard rise method, and the concentrated rinse method might generally show a higher sensitivity for detecting *Candida* in the oral cavity than the standard rinse method; therefore, we used results obtained via the concentrated rinse method rather than the standard rinse method for comparisons in the current study. Several sampling methods are available, including imprints, oral rinses, swabs, whole saliva collection [18], biopsies, and smears, and each method has both advantages and disadvantages [5]. Although the concentrated rinse method does not detect the localized site of infection, it enables quantitation of other microbes in addition to *Candida* species [5]. The concentrated rinse method is also easy to perform and is more sensitive than the imprint culture technique. Hence, it is suggested that the concentrated rinse method be preferentially employed in future investigations to obtain comparable data from different centers [8].

*Candida* counts may correspond to the severity of several clinical findings. Dry mouth was observed in 44 of 104 patients for whom *Candida* was detected by the concentrated rinse method, and the sensitivity, specificity, and positive predictive values of this characteristic were 42.3, 86.5, and 77.2 %, respectively. The *Candida* concentrations obtained using the concentrated rinse method showed some significant differences in the severity of dry mouth, redness of the tongue, residual root, coated tongue, and denture.

Similarly, the absence of a number of clinical signs (oral mucosa redness, angular cheilitis, residual root, glossalgia, taste disorder, denture, and ulceration) was a robust indicator for the absence of *Candida.* Similarly, low densities of *Candida* may not cause coated tongue, dry mouth, denture, redness of the tongue, and residual root, which are often observed in outpatients with *Candida* in the oral cavity; indeed, the *Candida* density showed a significant difference between the severities of each of these signs.

Taste disorder, redness of the oral mucosa, angular cheilitis, and ulceration were observed in less than 10 % of the outpatients diagnosed with candidiasis using the concentrated rinse method, and glossalgia was noted in 13.5 % of the outpatients diagnosed with candidiasis using the concentrated rinse method. In any case, all the above clinical oral signs were likely to be related to other oral diseases rather than to *Candida* infection. Concentrations of less than 90 CFU/100 μL obtained with the concentrated rinse method were not associated with any oral signs of candidiasis in outpatients and volunteers. The patients showing *Candida* colony numbers under 90 CFU/100 μL in the concentrated rinse method might have been in the stage before apparent candidiasis.

*Candida* species are often detected in the oral cavity in healthy individuals, and their presence does not necessarily indicate *Candida* infection. A threshold *Candida* concentration is required in order to separate individuals with commensal *Candida* from those with infection-associated *Candida*. Most healthy Thai adolescents carry *Candida* at a low level, that is, below 50 CFU/100 μL [19], and *Candida* levels of 60 CFU/100 μL in concentrated rinse culture samples are associated with healthy commensal carriage [8]. On the other hand, individuals with conditions that predispose them to infection harbor higher numbers (2 × 10^2^ to 3 × 10^2^ CFU/100 μL). *Candida* levels up to 9 × 10^2^ CFU/100 μL have been observed in healthy controls without clinical oral signs in other studies [7, 9].

Quantitative analysis may be important for the assessment of oral candidiasis, including differentiation from the commensal carriage of *Candida*. Oral candidiasis is a particularly significant problem with respect to the morbidity of immunocompromised individuals, including HIV-positive and AIDS patients, organ transplant recipients, and chemotherapy patients [10, 20, 21]. In addition, there have been several reports on the relationships between oral *Candida* and diabetes mellitus [22], oral *Candida* and Sjögren’s syndrome [23], and oral *Candida* and a combination of chronic renal failure and hemodialysis [24].

## Conclusions

In this study, the *Candida* concentration associated with several clinical oral signs in the infected patients and may be closely related to the patient’s current clinical status and prognosis. We have shown that quantitative analysis of *Candida* is required in order to correctly differentiate commensal forms of infection from those requiring treatment due to *Candida* infection. Such analysis may also be suitable for monitoring the time-dependent changes and quantitative analysis of *Candida* concentration. Adoption of the concentrated rinse method in independent locations around the globe is relatively straightforward since the method is simple. This will greatly facilitate direct comparisons between studies on *Candida* that originate in distinct geographic locations and involve diverse subject populations.

## Abbreviations

CFU: colony forming unit
SD: standard deviation

## References

[1] Axéll T, Samaranayake LP, Reichart PA, Olsen I (1997). A proposal for reclassification of oral candidosis. Oral Surg Oral Med Oral Pathol Oral Radiol Endod.

[2] Ghannoum MA, Jurevic RJ, Mukherjee PK, Cui F, Sikaroodi M, Naqvi A (2010). Characterization of the oral fungal microbiome (mycobiome) in healthy individuals. PLoS Pathog.

[3] Zegarelli DJ (1993). Fungal infections of the oral cavity. Otolaryngol Clin North Am.

[4] Sedgley CM, Samaranayake LP (1994). The oral prevalence of aerobic and facultatively anaerobic gram-negative rods and yeasts in Hong Kong Chinese. Arch Oral Biol.

[5] Williams DW, Lewis MA (2000). Isolation and identification of *Candida* from the oral cavity. Oral Dis.

[6] Byadarahally Raju S, Rajappa S (2011). Isolation and identification of *Candida* from the oral cavity. ISRN Dent.

[7] Samaranayake LP, MacFarlane TW, Lamey PJ, Ferguson MM (1986). A comparison of oral rinse and imprint sampling techniques for the detection of yeast, coliform and *Staphylococcus aureus* carriage in the oral cavity. J Oral Pathol.

[8] McKendrick AJ, Wilson MI, Main DM (1967). Oral *Candida* and long-term tetracycline therapy. Arch Oral Biol.

[9] White PL, Williams DW, Kuriyama T, Samad SA, Lewis MA, Barnes RA (2004). Detection of *Candida* in concentrated oral rinse cultures by real-time PCR. J Clin Microbiol.

[10] Saunus JM, Kazoullis A, Farah CS (2008). Cellular and molecular mechanisms of resistance to oral *Candida albicans* infections. Front Biosci.

[11] Hermann P, Berek Z, Nagy G, Kamotsay K, Rozgonyi F (2001). Pathogenesis, microbiological and clinical aspects of oral candidiasis (candidosis). Acta Microbiol Immunol Hung.

[12] Huskisson EC, Merzack R (1983). Visual analogue scales. Pain measurement and assessment.

[13] Collins SL, Moore RA, McQuay HJ (1997). The visual analogue pain intensity scale: what is moderate pain in millimeters?. Pain.

[14] Cancer Therapy Evaluation Program, Common Terminology Criteria for Adverse Events, Version 3.0, DCTD, NCI, NIH, DHHS, March 31, 2003, 2006, Aug 9. http://ctep.cancer.gov.

[15] Kakinoki Y, Nishihara T, Arita M, Shibuya K, Ishikawa M (2004). Usefulness of new wetness tester for diagnosis of dry mouth in disabled patients. Gerodontology.

[16] Eilers J, Berger A, Petersen M (1988). Development, testing, and application of the oral assessment guide. Oncol Nurs Forum.

[17] Kojima K (1985). Clinical studies on the coated tongue. Jpn J Oral Maxillofac Surg.

[18] Sugimoto J, Kanehira T, Mizugai H, Chiba I, Morita M (2006). Relationship between salivary histatin 5 levels and *Candida* CFU counts in healthy elderly. Gerodontology.

[19] Santiwongkarn P, Kachonboon S, Thanyasrisung P, Matangkasombut O (2012). Prevalence of oral *Candida* carriage in Thai adolescents. J Investig Clin Dent.

[20] Challacombe SJ, Naglik JR (2006). The effects of HIV infection on oral mucosal immunity. Adv Dent Res.

[21] Jain PA, Veerabhadrudu K, Kulkarni RD, Ajantha GS, Shubhada C, Amruthkishan U (2010). Comparative study of adherence of oral *Candida albicans* isolates from HIV sero-positive individuals and HIV sero-negative individuals to human buccal epithelial cells. Indian J Pathol Microbiol.

[22] Soysa NS, Samaranayake LP, Ellepola AN (2006). Diabetes mellitus as a contributory factor in oral candidosis. Diabet Med.

[23] Yan Z, Young AL, Hua H, Xu Y (2011). Multiple oral *Candida* infections in patients with Sjogren’s syndrome -- prevalence and clinical and drug susceptibility profiles. J Rheumatol.

[24] Godoy JS, de Souza Bonfim-Mendonça P, Nakamura SS, Yamada SS, Shinobu-Mesquita C, Pieralisi N (2013). Colonization of the oral cavity by yeasts in patients with chronic renal failure undergoing hemodialysis. J Oral Pathol Med.

